# Microbes Producing L-Asparaginase free of Glutaminase and Urease isolated from Extreme Locations of Antarctic Soil and Moss

**DOI:** 10.1038/s41598-018-38094-1

**Published:** 2019-02-05

**Authors:** Anup Ashok, Kruthi Doriya, Jyothi Vithal Rao, Asif Qureshi, Anoop Kumar Tiwari, Devarai Santhosh Kumar

**Affiliations:** 1 Industrial Bioprocess and Bioprospecting Laboratory (IBBL), Department of Chemical Engineering, Indian Institute of Technology Hyderabad, Kandi, Sangareddy, Telangana State 502285 India; 2 Emerging Contaminants Group (ECG), Department of Civil Engineering, Indian Institute of Technology Hyderabad, Kandi, Sangareddy, Telangana State 502285 India; 3 National Centre for Polar and Ocean Research (NCPOR), Vasco da Gama, Goa, 403804 India

## Abstract

L-Asparaginase (L-asparagine aminohydrolase, E.C. 3.5.1.1) has been proven to be competent in treating Acute Lymphoblastic Leukaemia (ALL), which is widely observed in paediatric and adult groups. Currently, clinical L-Asparaginase formulations are derived from bacterial sources such as *Escherichia coli* and *Erwinia chrysanthemi*. These formulations when administered to ALL patients lead to several immunological and hypersensitive reactions. Hence, additional purification steps are required to remove toxicity induced by the amalgamation of other enzymes like glutaminase and urease. Production of L-Asparaginase that is free of glutaminase and urease is a major area of research. In this paper, we report the screening and isolation of fungal species collected from the soil and mosses in the Schirmacher Hills, Dronning Maud Land, Antarctica, that produce L-Asparaginase free of glutaminase and urease. A total of 55 isolates were obtained from 33 environmental samples that were tested by conventional plate techniques using Phenol red and Bromothymol blue as indicators. Among the isolated fungi, 30 isolates showed L-Asparaginase free of glutaminase and urease. The L-Asparaginase producing strain *Trichosporon asahii* IBBLA1, which showed the highest zone index, was then optimized with a Taguchi design. Optimum enzyme activity of 20.57 U mL^−1^ was obtained at a temperature of 30 °C and pH of 7.0 after 60 hours. Our work suggests that isolation of fungi from extreme environments such as Antarctica may lead to an important advancement in therapeutic applications with fewer side effects.

## Introduction

Acute Lymphoblastic Leukaemia (ALL) is the most common type of childhood cancer. One of the major methods to treat ALL is through the enzyme L-Asparaginase. L-Asparaginase diminishes the supply of asparagine to cancer cells, thereby leading to apoptosis and cell cycle arrest in the G1 phase^1,2^. However, depletion of glutamine due to the dual glutaminase and L-Asparaginase activity may lead to pancreatitis, haemostasis abnormalities, central nervous system dysfunction and immunological reactions due to antibody production^3,4^. Recent breakthroughs have made asparaginase treatment on children and young adults more feasible. The administration of this chemotherapeutic drug in adults over the age of 45 years has shown tolerance but at the cost of higher toxicity^5^. Additionally, presence of urease activity has been detected in commercial preparations, which can also reduce the efficacy of ALL treatment^6^. A detailed description of the effect of glutaminase and urease on the outcome of L-Asparaginase activity is given in supplementary data. Apart from medicinal applications, L-Asparaginase is also used in the food industry to prevent acrylamide formation at high temperatures, which otherwise has carcinogenic and neurotoxic implications^7,8^.

Currently, L-Asparaginase is industrially produced mainly from prokaryotic microbes such as *Escherichia coli* and *Erwinia chrysanthemi*. However, the concomitant presence of the activities of glutaminase and urease means that additional purification steps are required to remove these two enzymes and maintain drug efficacy^9,10^. The presence of glutaminase destroys the necessary glutamine required for the growth of normal cells^11^. Urease leads to the hydrolysis of the urea content present in the blood, which is toxic^12^. The key focus in the past has been on the isolation of cultures that produce L-Asparaginase that is free of glutaminase^13,14^ whereas here we aim to isolate cultures that produce L-Asparaginase free of glutaminase as well as urease. L-Asparaginase produced from eukaryotic microorganisms such as fungi has been suggested as an alternative source that has fewer side effects^15,16^. In particular, L-Asparaginase that is free of glutaminase and urease activity will be most beneficial. Because of their eukaryotic nature, fungal species have the capacity to replicate the effects of human cells and can be used in the treatment of cancer with better success than other microorganisms^17^. Here, we investigate the efficiency of fungal species isolated from the terrestrial environment of Antarctica in producing such an enzymatic product.

Antarctica is one of the coldest and driest places on earth. Salient environmental characteristics are frequent freeze-thaw cycles, low nutrient availability, high ultraviolet radiation and prolonged periods of continuous light and darkness^18,19^. In spite of these harsh conditions, many microbes remain viable. A major ecological role is played by fungi, which influence continental primary production by being mycobiont partners in linchen symbiosis^20^. They are key organisms involved in the biodegradation of organic matter, thereby influencing the carbon and nitrogen cycles. The most common fungal species reported in Antarctica are *Basidiomycota*, *Ascomycota*, and *Zygomycota phyla*^21^.

Fungi (yeasts) in Antarctica are both indigenous and exogenous (brought to the continent by ocean and wind currents and the migration of birds and humans). All these species seem to have adapted to the extreme conditions of Antarctica. Known mechanisms of adaptation include secretion of anti-freeze proteins and higher enzyme activity at lower temperatures^22^. Peptides and glycopeptides of various sizes decrease the freezing point of water by binding to ice crystals. Thermodynamically, reactions catalysed by psychrophilic enzymes are characterized by low free energy of activation and a low enthalpy change. Active sites are larger and more accessible to substrates^23,24^. As a result, these active sites are less stable, and the enzymes are up to ten times more active than mesophilic enzymes at temperatures up to 20–30 °C. As such, enzymes from psychrophiles have immense potential in biotechnological applications^25^.

Here, we investigate whether such enzymes from cold-adapted Antarctic eukaryotes (fungi) may have potential applications in producing L-Asparaginase free from glutaminase and urease. We analyse 55 isolates obtained from 33 environmental samples from soil, moss, water and ice collected from the Schirmacher Hills (latitude 70°43′50″S–70°46′40″S and longitude 11°22′40″E–11°54′25″E), Dronning Maud Land, Antarctica, using conventional plate techniques. Alternative sources like the Canadian Arctic, Siberia, and Greenland can also be investigated for similar strains. Zone index is calculated to obtain a semi-quantitative assessment of the activity of enzymes in these isolates, that helps in the primary analysis of the species. DNA sequencing is done to identify the five most promising cultures. Operational parameters to achieve a higher enzyme activity in the most promising isolate are optimized using the Taguchi Orthogonal Array (OA) technique.

## Materials

### Site description

**Figure 1:**
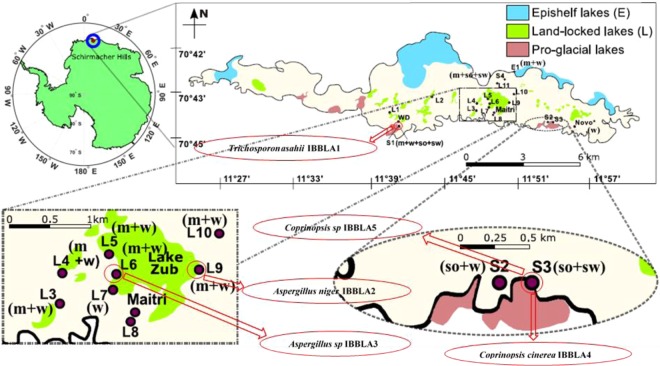
Sampling site locations [media collected at any site: m = moss, w = water, so = soil, sw = snow]. Five dominant fungal species which showed highest zone index and their locations are shown in oval boxes.

Schirmacher Hills, Antarctica, is an exposed bedrock that is situated in an area of about 35 km^2^ bounded by ice shelves in the north and continental ice sheets in the south^26^. The region consists of over 105 freshwater lakes^27^. These lakes have low mineral content as they are approximately 80 km away from the coastal region. Microbes are the primary stage growth factors for further complex growth in the region^18^. The region hosts two research stations, Maitri (Indian, Est. 1989) and Novolazarevskaya (Russian, Est. 1961), and a tourist ‘campsite’ (White Desert, Est. 2006). Sampling locations are shown in Fig. 1.

## Methods

### Laboratory analysis

#### Semi-quantitative assay for L-Asparaginase producing fungi

Primary analysis of the Antarctic samples was done using Modified Czapek Dox (MCD) medium using phenol red as an indicator. The composition of the prepared MCD medium was: glucose 2 g L^−1^, L-asparagine or glutamine or filter sterilized urea 10 g L^−1^, KH_2_PO_4_ 1.52 g L^−1^, KCl 0.52 g L^−1^, MgSO_4_.7H_2_O 0.52 g L^−1^, trace amounts of FeSO_4_.7H_2_O, ZnSO_4_.7H_2_O and CuNO_3_.3H_2_O and agar 18 g L^−1 ^^28^. About 2.5% (w/v) stock solution of the phenol red dye was prepared and MCD medium was supplemented with 0.009% phenol red dye. The final pH of the medium was adjusted to 5.5 using 1 M NaOH^29^. The prepared medium was then autoclaved, and poured into pre-sterilized plates. Control plates were prepared with NaNO_3_ as the sole nitrogen source. MCD plates were inoculated with isolated fungi as test organism and *A. terreus* MTCC 1782 as a positive test^30,31^. The colony diameter and zone diameter for all the test organisms were calculated by measuring the inner and outer diameter of the microorganisms’ growth and enzyme production respectively after 96 h of incubation. Zone index was calculated as the ratio of outer to inner diameter as shown in Equation 1.

1 $$Zone\,Index=\frac{Zone\,diameter}{Colony\,diameter}$$

These semi-quantitative results were further verified using bromothymol blue (BTB). For this, 0.04% (w/v) of stock solution of the bromothymol blue dye was prepared and 0.007% BTB dye was supplemented in MCD medium. The remaining procedure was the same as for phenol red.

#### Identification of the fungal species

Morphological observation of positive isolates was done by the method of staining and observing fungal spores using lacto-phenol cotton blue staining solution.

For DNA identification of the five strains that showed the five highest zone indices, about 100 mg of wet fungal tissue was re-suspended in 100 µL of Extraction buffer. An equal volume of 0.5 mm glass beads was added and vigorously vortexed for 10 minutes. The lysed sample was centrifuged and the supernatant collected to a fresh microfuge tube. DNA was precipitated from the supernatant by using alcohol and the precipitated DNA was washed with 70% alcohol, air dried and re-suspended in Tris-EDTA buffer. The DNA was quantified and checked on Agarose gel for integrity. 100 ng of DNA was used in PCR using standard primers for the amplification of ITS 1, 5.8S ribosomal RNA gene and ITS2 region.

The primer set ITS 1 and ITS 4^32^ did not show any amplification but primer sets ITS 1 F and ITS 4 R/ITS4B^33^ showed good amplification. The PCR products were purified by gel elution and the purified product was sequenced using Sanger dideoxy method on ABI 3130 (48 capillary) or 3730Xl (96 capillary) electrophoresis instruments by BioArtis India Pvt Ltd.

#### Quantitative estimation of L-Asparaginase activity in selected strains

L-Asparaginase activity was quantified in the five isolates that produced the five highest zone indices. These isolates were cultivated on potato dextrose slants at 30 °C for 96 h. From these slants, 1 mL of suspension was inoculated into Erlenmeyer flasks containing 50 mL of MCD medium with initial pH of 6.2. The flasks were incubated at 30 °C at 180 rpm for 96 h. Samples were withdrawn every 12 h for the extraction of enzyme and determination of L-Asparaginase activity.

The L-Asparaginase activity was measured as the amount of ammonia released during the enzyme reaction using Nesslerization method^34–36^. The crude enzyme sample was extracted by centrifugation and supernatant that contains enzyme was used for analysis.

Two sets of samples were prepared in triplicates, one set named as “Test” and the other set as “Blank”. Both these samples contained 900 µL of the Tris-HCl buffer and L-Asparagine mixture at pH 8.6. Crude enzyme supernatant (100 µL) was added to the Test sample and kept for reaction at 37 °C for 30 min. After 30 min the reaction was quenched by adding 1.5 M TCA (Trichloroacetic acid) solution. For the blank, supernatant was stored at 4 °C and the reaction was terminated with TCA first followed by the addition of crude enzyme.

These samples were then centrifuged to remove any particles that might have been formed during the reaction. An amount of 100 µL of the re-centrifuged supernatant (test and blank) was added to tubes containing 3.8 mL of water followed by Nessler’s reagent as indicator and kept for 15 min for the complete sample to stabilize. The spectrophotometric analysis was done at 425 nm wavelength for both Test and Blank samples and the difference between both these values was described as the amount of ammonia liberated by the enzyme due to its reaction with the L-asparagine content as shown in Supplementary Fig. S3. All the analysis was done in triplicate and therefore, results are represented as their average values. Moreover, the amount of free ammonia present in the initial supernatant was also calculated and the values obtained were similar to the ones obtained in the Blank samples. A standard curve was prepared using ammonium sulphate to determine the activity with respect to the ammonia liberated in the reaction. Enzyme activity was calculated by the amount of ammonia released using a standard graph^37^ as shown in Equation 2.

2 $${\rm{Enzyme}}\,{\rm{Activity}}\,({\rm{U}}\,{{\rm{mL}}}^{-1})=\frac{{\rm{Amount}}\,{\rm{of}}\,{{\rm{NH}}}^{4+}\,{\rm{liberated}}}{{\rm{incubation}}\,{\rm{time}}\times {\rm{mL}}\,{\rm{of}}\,{\rm{enzyme}}\,{\rm{taken}}}$$

The glutaminase and urease activities were determined by using the similar method of Nesslerization which was used to determine the activity of L-asparaginase. The buffer mixture containing L-asparagine-Tris-HCl was replaced with L-glutamine- Tris-HCl and urea- Tris-HCl respectively and the same procedure as described above was used to determine the enzyme activity. The data for the enzyme activities are shown in the Supplementary Data Fig. S4.

#### Optimization of nutrient and culture conditions

Based on initial quantitative estimation of the enzyme activity, Taguchi Orthogonal Array method with four different parameters (temperature, pH, L-Asparagine concentration and Glucose concentration)^38^ was selected and three different levels for each of these parameters were applied (Table 1). A detailed insight into the optimization methodology is given in the supplementary data

**Table 1:** Factors and the range of the values used in the statistical analysis using Taguchi Orthogonal Array method.

Factor	Name	Units	Minimum	Maximum	Level of factors
A	Temperature	°C	25	35	3
B	pH		6	8	3
C	L-Asparagine	g L^−1^	9	11	3
D	Glucose	g L^−1^	1	3	3

Among all the strains tested, *Trichosporon asahii* IBBLA1 showed highest enzyme activity. To further enhance the culture conditions, a total of 9 experiments were conducted on this isolate. Analyses was done using Design Expert version 9.0 (Stat-Ease Inc., Minneapolis, USA). Cultivation parameters according to the design matrix and their respective responses on L-Asparaginase activity are shown in Table 2.

**Table 2:** Experimental conditions and the output enzyme activity result from each of the runs specific to the Taguchi Orthogonal Array method.

S.No	Run	Temperature (°C)	pH	L-Asparagine (g L^−1^)	Glucose (g L^−1^)	Activity (U mL^-1^)
1	4	25	6	9	1	5.34
2	2	25	7	10	2	6.94
3	9	25	8	11	3	3.94
4	8	30	7	10	2	20.57
5	3	30	8	11	3	15.29
6	7	30	6	9	1	16.93
7	6	35	8	11	3	6.35
8	1	35	6	9	1	7.64
9	5	35	7	10	2	9.25

## Results and Discussions

**Figure 2:**
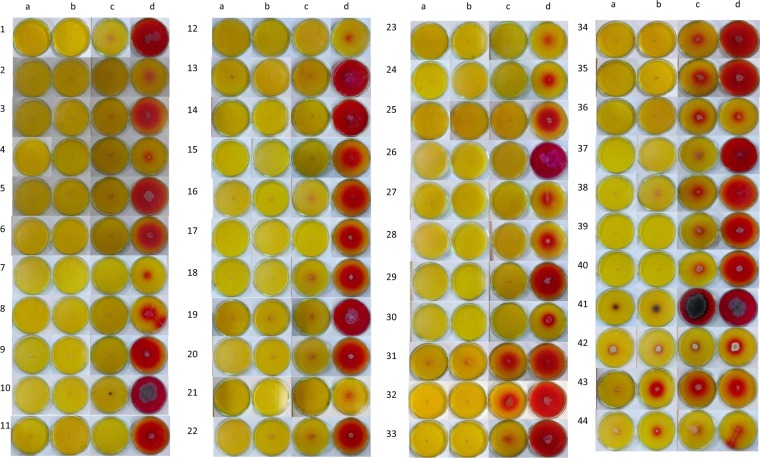
Isolation and screening for L-Asparaginase producing micro-organism with different nitrogen source, on plate supplemented with phenol red dye. ‘a’ indicates the isolate grown on plate containing NaNO_3_ as sole nitrogen source. ‘b’ indicates the isolate grown on plate containing urea as a sole nitrogen source. ‘c’ indicates the isolate grown on plate containing glutamine as a sole nitrogen source. ‘d’ indicates the isolate grown on plate containing L-Asparagine as a sole nitrogen source. Table 3 contains the list of the samples. Numbers 1–30 are isolates that produced L-Asparaginase free of glutaminase and urease, numbers 30–41 produced L-Asparaginase and glutaminase free of urease, numbers 41–44 produced all of L-Asparaginase, glutaminase and urease.

**Figure 3:**
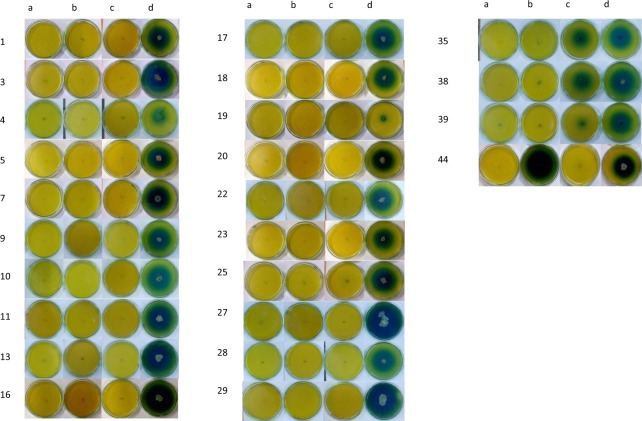
Isolation and screening for L-Asparaginase producing micro-organism with different nitrogen source, on plate supplemented with BTB dye. ‘a’ indicates the Isolate grown on plate containing NaNO_3_ as sole nitrogen source. ‘b’ indicates the Isolate grown on plate containing urea as a sole nitrogen source. ‘c’ indicates the Isolate grown on plate containing glutamine as a sole nitrogen source. ‘d’ indicates the Isolate grown on plate containing L-Asparagine as a sole nitrogen source. Table 3 contains the list of the samples. Numbers 1–29 are isolated that produced L-Asparaginase free of glutaminase and urease, 35, 38 and 39 produced L-Asparaginase and glutaminase free of Urease, and 44 produced L-Asparaginase and urease free of glutaminase. The remaining isolated did not produce any of the enzymes.

A total of 55 isolates were obtained from 33 samples out of which 41 were obtained from moss samples and 14 from soil samples (Figs 2 and 3).

### Primary screening analysis using Phenol Red Indicator

Out of the 55 obtained isolates, 44 isolates showed growth in different substrate media. Thirty of these 44 showed growth in asparagine media only, while 11 showed growth in glutamine media along with asparagine media and 3 showed growth in all the substrate media (i.e. asparagine, glutamine and urea). Eleven isolates did not show affinity to any of the substrate media. The maximum value of zone index obtained was 5.8 for isolate which produced L-Asparaginase free of glutaminase and urease, and 8.0 for isolate which produced urease free L-Asparaginase and glutaminase. The zone indices of all isolates obtained are given in the Table 3.

**Table 3:** Zone index values for the isolates obtained from the Antarctica samples using Phenol red and BTB as indicators representing their extent of crude enzyme production at 96 h.

Sl. No	Sample Name-Sample #	Location	Sample Nature	Phenol Red Indicator				Bromothymol Blue indicator			
				Asn	Gln	Urease	NaNO_3_	Asn	Gln	Urease	NaNO_3_
1	E1–1	70°45′04.20″S 11°44′37.30″E	Moss	5.00	—	—	—	—	—	—	—
2	E1–2		Moss	1.00	—	—	—	—	—	—	—
3	L3-1	70°45′52.94″S 11°42′35.64″E	Moss	4.30	—	—	—	—	—	—	—
4	L3-2		Moss	2.00	—	—	—	—	—	—	—
5	L4-1	70°45′44.60″S 11°42′43.10″E	Moss	3.65	—	—	—	4.14	—	—	—
6	L4-2		Moss	3.33	—	—	—	—	—	—	—
7	L4-3		Moss	2.00	—	—	—	—	—	—	—
8	L4-4		Moss	4.54	1.20	—	—	—	—	—	—
9	L5-1	70°45′47.60″S 11°49′44.60″E	Moss	2.00	—	—	—	—	—	—	—
10	L5-2		Moss	3.71	—	—	—	2.85	—	—	—
11	L6-1	70°45′51.90″S 11°49′29.80″E	Moss	4.76	—	—	—	7.60	—	—	—
12	L6-2		Moss	4.50	—	—	—	—	—	—	—
13	L6-3		Moss	4.39	1.42	—	—	5.40	1.20	—	—
14	L6-4		Moss	4.44	1.60	—	—	4.0	3.0	—	—
15	L6-5		Moss	3.88	1.66	—	—	—	—	—	—
16	L9-1	70°45′16.70″S 11°50′20.40″E	Moss	4.68	—	—	—	5.18	—	—	—
17	L9-2		Moss	1.49	—	—	—	3.80	—	—	—
18	L9-3		Moss	6.73	2.73	—	—	—	—	—	—
19	L9-4		Moss	2.01	1.53	—	—	—	—	—	—
20	L10-1	70°45′38.60″S 11°51′25.86″E	Moss	2.53	1.00	1.00	—	—	—	—	—
21	S1-1	70°45′56.10″S 11°37′7.41″E	Soil	4.80	—	—	—	—	—	—	—
22	S1-2		Moss	3.67	—	—	—	4.00	—	—	—
23	S1–3		Soil	8.00	3.33	—	—	3.42	2.66	—	—
24	S1–4		Moss	3.83	—	—	—	4.71	—	—	—
25	S1–5		Moss	1.50	—	—	—	2.6	—	—	—
26	S1–6		Moss	3.18	—	—	—	4.0	—	—	—
27	S1–7		Moss	3.50	1.20	—	—	—	—	—	—
28	S2-1	70°46′23.20″S 11°47′35.00″E	Soil	2.50	—	—	—	2.85	—	—	—
29	S2-2		Soil	1.85	1.29	—	—	—	—	—	—
30	S2–3		Soil	2.50	2.41	5.71	—	—	—	—	—
31	S3-1	70°46′23.75″S 11°48′4.60″E	Soil	3.61	—	—	—	3.75	—	—	—
32	S3-2		Soil	3.45	—	—	—	3.33	—	—	—
33	S3-3		Soil	2.81	—	—	—	—	—	—	—
34	S3–4		Soil	4.92	—	—	—	5.33	—	—	—
35	S3–5		Soil	5.83	—	—	—	4.00	—	—	—
36	S3–6		Soil	5.68	—	—	—	4.25	—	—	—
37	S3–7		Soil	3.50	—	—	—	3.14	—	—	—
38	S3–8		Soil	2.18	2.00	2.00	—	3.62	—	11.8	—
39	S4-1	70°45′16.04″S 11°44′58.25″E	Soil	2.20	—	—	—	2.88	—	—	—
40	S4-2		Moss	5.00	—	—	—	4.80	—	—	—
41	S4-3		Moss	4.00	—	—	—	3.00	—	—	—
42	S4-4		Moss	4.77	—	—	—	5.42	—	—	—
43	S4–5		Moss	4.24	1.64	—	—	—	—	—	—
44	S4–5		Moss	4.24	1.75	—	—	—	—	—	—

41 isolates were observed from moss samples, only 30 have shown production of any of the enzyme and these are numbered as follows- S1 (22, 24, 25, 26, 27), L5 (9, 10), S4 (40, 41, 42, 43, 44), L6 (11, 12, 13, 14, 15), L3 (3, 4), L-10 (20), E1 (1, 2), L9 (16,17,18,19) L4 (5, 6, 7, 8);14 isolates were observed from soil samples and all have shown production of any of the enzyme and these are numbered as follows- S1 (21, 23), S4 (39), S2 (28, 29, 30), S3 (31, 32, 33, 34, 35, 36, 37, 38); ‘—’

indicates no growth was observed in that media.

### Confirmatory screening analysis using BTB Indicator

Zone index values of isolates using BTB dye were similar to the results obtained using phenol red. Out of the 34 isolates produced, 28 showed positive growth of L-Asparaginase free of glutaminase and urease. BTB showed a better zone of hydrolysis whereas plates containing phenol red dye showed less contrast (Figs 2 and 3). Maximum zone index value in BTB indicator is 7.6 for isolates which produced L-Asparaginase free of glutaminase and urease, 5.4 for isolates which produced L-Asparaginase and glutaminase free of urease, 3.62 for isolates which produced L-Asparaginase and urease free of glutaminase (Table 3).

### Identification of five most promising cultures and quantification of their enzyme activity

**Figure 4:**
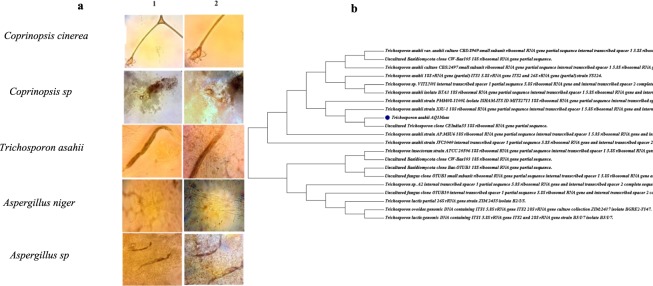
(**a**) Microscopic images of five isolates that exhibited the highest zone indices and produced L-Asparaginase free of glutaminase and urease [taken using CETI Max II Binocular Microscopes 1202.4000 M]. Column 1: 40x magnification, Column 2: 100x magnification. (**b**) the phylogenetic tree of *Trichosporon asahii* IBBLA1, the microbe with highest enzyme activity, based on 5.8SrRNA for strain S1–3.

Microscopic images (Fig. 4, 40x and 100x magnification) for all five cultures, along with the phylogenetic tree for the culture with the highest enzyme activity suggests that the isolates obtained from the samples are mostly fungal or actinomycetes in nature. Further confirmation of the genome species was done through gene squencing. The dominant species were *Coprinopsis* sp., *Trichosporon sp*., and *Aspergillus sp*. The ITS rRNA sequences of *Trichosporon asahii* IBBLA1 *and Coprinopsis cinerea* IBBLA4 were deposited in NCBI GenBank under the accession number MH443321 and MH443753, respectively.

**Figure 5:**
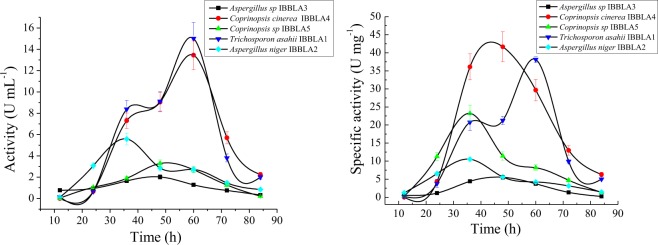
L-Asparaginase activity (**a**) and specific activity (**b**) of the selected strains.

*Trichosporon asahii* IBBLA1 and *Coprinopsis cinerea* IBBLA4 species showed enzyme activity of 15 U mL^−1^ and 13.5 U mL^−1^, respectively (Fig. 5), using One-factor- at-a-time technique. Maximum L-Asparaginase activity was obtained at 60 h during the fermentation process. A quantitative assessment for the production of glutaminase and urease was also done^33^. Glutaminase and urease activities were not significant in comparison to the L-Asparaginase activity as shown in Supplementary Fig. S4. The effect of other parameters like inoculum concentration is also shown in Supplementary Fig. S2a,b. Operational conditions for the sample producing the highest enzyme activities *Trichosporon asahii* IBBLA1 and *Coprinopsis cinerea* IBBLA4 were then optimized using the Taguchi method.

### Optimization of L-Asparaginase production using Taguchi method

**Figure 6:**
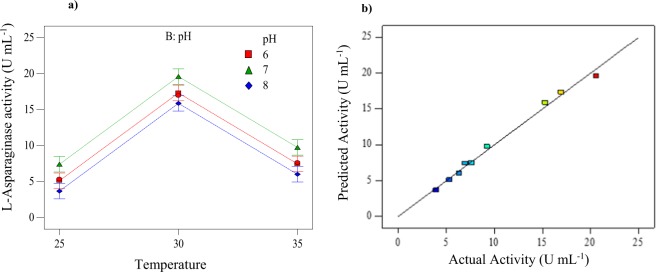
(**a**) Interaction plot between the two primary factors of temperature and pH showing the variation in the production of the enzyme. (**b**) Taguchi model predicted versus experimentally obtained actual L-Asparaginase enzyme activity.

Production of L-Asparaginase varied from 3.94 to 20.57 U mL^−1^ (Table 2) for *Trichosporon asahii* IBBLA1 which denotes that the factors used for the Taguchi method had a considerable impact on the enzyme production. The interactions between the primary parameters, obtained from two-way ANOVA, are shown in Fig. 6a and a comparison of actual results and the Taguchi predicted model is shown in Fig. 6b. Equation 3 is developed that relates the L-Asparaginase activity individual parameters

3 $${\rm{L}} \mbox{-} \mathrm{Asparaginase}\,{\rm{Activity}}=10.25-4.843\times {x}_{1}+7.346\times {x}_{2}-0.28\times {y}_{1}+2.0\times {y}_{2}$$

The coefficients are selected based on the *F* and *p*-values as in Table 4, the ‘*x*_1_ and *x*_2_’ values correspond to the coded temperature data, and the ‘*y*_1_ and *y*_2_’ values correspond to the coded pH data. Coded value is equal to the ratio: [(Actual value − mean)/(range/2)]. When the values are replaced with experimental pH and temperature data, equation 3 computes a different value of L-Asparaginase activity.

**Table 4:** Two-way ANOVA analysis of the main effects with coefficients that help in determining the significance of each parameter and also the model efficiency.

Source	Sum of squares	Degrees of freedom	Mean square	*F* value	*p*-value Probability > *F*
Model	272.28	4	68.07	126.66	0.0002
A-Temperature	251.0942	2	125.55	233.6	0.0001
B-pH	21.18	2	10.59	19.71	0.0085
C-L-Asparagine	0	0	—	—	—
D-Glucose	0	0	—	—	—
Residual	2.15	4	0.54	—	—
Corrected total	274.4288	8	—	—	—

The regression coefficient (*R*^2^) of this model was 0.99 indicating that only 1% of the variability in data cannot be explained by the Taguchi model. The signal to noise ratio for the model is expected to be above 4.0 and the value obtained in the current study is 29.129. The predicted and adjusted *R*^2^ values are approximately equal. The *F* and *p-*values along with the above observations confirm that the chosen model is significant. The operational parameters which produced the highest enzyme activity (20.57 U mL^−1^) are: a temperature of 30 °C, a neutral pH, L-Asparagine concentration of 10 g L^−1^ and a Glucose concentration 2 g L^−1^. This enzyme activity is much higher compared to those obtained using some other bacterial techniques (2–5 U mL^−1^) which are being used currently^39,40^ and comparable to others obtained under similar laboratory conditions^41,42^. The optimization of *Coprinopsis cinerea* IBBLA4 has been shown in the supplementary data with the corresponding tables (Supplementary Tables S1–S3) and figures (Supplementary Fig. S1). The fungal species provide the added advantage of the absence of glutaminase and urease activities. In comparison to the similar fungal species the activity obtained in our study is comparatively higher, making it a potential source for the production of the enzymes for its unhindered use in production of commercial anti-cancer drugs without the bacterial side-effects^43^.

## Conclusions

A selective multi-screening method was used for isolating L-Asparaginase producing microorganism from the Schirmacher Hills region of Antarctica. A total of 30 among the 55 isolates produced L-Asparaginase free of glutaminase and urease. Microscopic images and DNA sequencing of the five isolates that produced the highest zone indices confirmed that they are fungal in nature. Operational conditions were optimized for the isolate which showed highest enzyme activity using Taguchi method, and the optimized model was found to be significant (*R*^2^ = 0.99). Maximum enzymatic activity of 20.57 U mL^−1^ was exhibited by *Trichosporon asahii* IBBLA1 at a temperature of 30 °C, pH of 7.0, and L-Asparagine and glucose concentrations of 10 g L^−1^ and 2 g L^−1^, respectively. Obtained enzyme activity is comparable to that from bacterial sources. Our results show promise towards development of techniques suitable for the large scale production of L-Asparaginase that will be free of any glutaminase or urease content, which is currently being pursued in our lab. Fungal species have the ability to mimic the properties of the human cells, as both are eukaryotic in nature, which makes it easier for their usage in treatment of ALL. We find that extremophiles may be particularly helpful in the treatment of ALL and in the food industry.

## Supplementary information

Supplementary Information
