# Supplementary material for: Microbes Producing L-Asparaginase free of Glutaminase and Urease isolated from Extreme Locations of Antarctic Soil and Moss
Source: Sci Rep. 2019 Feb 5;9:1423. doi: 10.1038/s41598-018-38094-1 (PMC6363723; doi:10.1038/s41598-018-38094-1)
Supplement: Supplementary file 1 — Supplementary Information [file 41598_2018_38094_MOESM1_ESM.docx]

**Microbes Producing L-Asparaginase free of Glutaminase and Urease isolated from Extreme Locations of Antarctic Soil and Moss**

Anup Ashok^1^, Kruthi Doriya^1^, Jyothi Vithal Rao^1^, Asif Qureshi^2^, Anoop Kumar Tiwari^3^, & Devarai Santhosh Kumar^1^*

^1^Industrial Bioprocess and BioProspecting Laboratory (IBBL), Department of Chemical Engineering, Indian Institute of Technology Hyderabad, Kandi, Sangareddy, Telangana State - 502285, India.

^2^Department of Civil Engineering, Indian Institute of Technology Hyderabad, Kandi, Sangareddy, Telangana State - 502285, India.

^3^National Centre for Polar and Ocean Research (NCPOR), Vasco da Gama, Goa - 403804, India.

*corresponding author

Dr Devarai Santhosh Kumar

Assistant Professor

Department of Chemical Engineering

IIT Hyderabad

Kandi, Sangareddy, TS – 502285, India.

Email: devarai@iith.ac.in

Ph.: +91 4023017122; + 91 4023016156

**Supplementary Information**

The Leukemic cells are deprived of the enzyme asparagine synthetase, which is *de novo* synthesized in normal cells, hence these cancer cells attack the asparagine present in blood serum for their growth and survival. If the leukemic cells can be devoid of the asparagine content, then their growth can be curtailed. The L-Asparaginase specifically targets these asparagine content present within the cells and then hydrolyzes it to aspartic acid and ammonia, thereby limiting the growth of neoplastic cells. A schematic representation is shown below.

Glutamine is another essential amino acid that is considered as building block of proteins present in the body, it plays a major role in maintaining immunity of the body along with many other functions. It helps in the growth and proper functioning of the stomach and intestinal cells. Glutamine is the precursor of the enzyme asparagine synthetase. The presence of L-glutaminase causes the breakdown of these glutamine content leading to several immunological reactions such as Pancreatitis, Hepatotoxicity, Neurotoxicity. A representation of the glutaminase activity is shown below.

L-glutamine

L-glutamic acid

H_2_O

NH_3_

L-glutaminase

Urea is the most common metabolic waste that is produced in the body, it can be removed in the form of urine as it is a highly soluble organic compound. It is formed from ammonia as a part of deamination of the amino acids in the liver. Urease is an enzyme that catalyzes the hydrolysis of urea and produces ammonia and carbon dioxide. The ammonia that was being removed from the blood stream as urea is thereby converted back to ammonia leading to a condition called Hyperammonemia which causes brain injury. The ammonia that is released in this process is twice the amount released in the other processes. A schematic of the process is given below

Urea

CO_2_

H_2_O

2NH_3_

Urease

**Zone index**


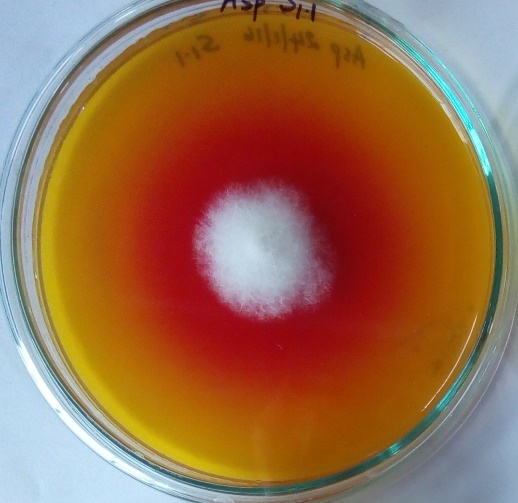


b

a

The figure shows the growth criteria of the microorganism which gives the indication to the zone index calculations. The terms depicted are colony diameter (a) and zone diameter (b). Colony diameter indicates the diameter of the growth of the microorganism while the zone diameter depicts the extent of enzyme activity based on the change in color due to the change in the pH.

$$Zone Index=\frac{Zone diameter}{Colony diameter}=\frac{b}{a}$$

It has been shown that there exists a direct relation between the value on zone index and enzyme activity in different culture broths. The values of different zone index values have been shown in Table 3 of the main manuscript file.

The interactions between the primary parameters, obtained from two-way ANOVA, are shown in Figure S1a and a comparison of actual results and the Taguchi predicted model is shown in Figure S1b. An equation is developed that relates the L-Asparaginase activity individual parameters

$$\text{L-Asparaginase Activity}=8.36-3.07\times x_{1}+4.26\times x_{2}-0.39\times y_{1}+2.32\times y_{2}$$

The coefficients are selected based on the *F* and *p*-values as in Table S3 and the ‘*x*_1_ and *x*_2_’ values corresponds to the coded temperature data and the ‘*y*_1_ and *y*_2_’ values corresponds to the coded pH data. Coded value = [(Actual value - mean)/(range/2)]. When the values in coding formulas are replaced with experimental pH and temperature data, the equation computes different values of L-Asparaginase activity.

The regression coefficient (*R^2^*) of this model was 0.9664 indicating that only 3.36% of the variable in data cannot be explained clearly by the Taguchi model. The signal to noise ratio for the model is expected to be above 4.0 and the value obtained in the current study is 15.566. The predicted and adjusted R^2^ values are approximately equal. The *F-* and *p-*values along with the above observations confirm that the chosen model is significant. The operational parameters that produced the highest enzyme activity (16.23 U mL^-1^) are: a temperature of 30ºC, a neutral pH, L-Asparagine concentration of 9 g L^-1^ and a Glucose concentration 2 g L^-1^

**Supplementary Table S1.** Factors and the range of the values used in the statistical analysis using Taguchi Orthogonal Array method for *Coprinopsis cinerea* IBBLA4.

| Factor | Name | Units | Minimum | Maximum | Level of factors |
| --- | --- | --- | --- | --- | --- |
| A | Temperature | ºC | 25 | 35 | 3 |
| B | pH |  | 6 | 8 | 3 |
| C | L-Asparagine | g L^-1^ | 9 | 11 | 3 |
| D | Glucose | g L^-1^ | 1 | 3 | 3 |

**Supplementary Table S2.** Experimental conditions and the output enzyme activity result from each of the runs specific to the Taguchi Orthogonal Array method for *Coprinopsis cinerea* IBBLA4.

| **S.No** | **Run** | **Temperature**  **(ºC)** | **pH** | **L-Asparagine**  **(g L^-1^)** | **Glucose**  **(g L^-1^)** | **Activity**  **U mL^-1^** |
| --- | --- | --- | --- | --- | --- | --- |
| 1 | 1 | 25 | 7 | 2 | 10 | 6.70 |
| 2 | 2 | 35 | 7 | 1 | 11 | 9.08 |
| 3 | 3 | 25 | 8 | 3 | 11 | 3.81 |
| 4 | 4 | 30 | 7 | 2 | 9 | 16.23 |
| 5 | 5 | 30 | 8 | 1 | 10 | 9.96 |
| 6 | 6 | 35 | 6 | 3 | 10 | 6.91 |
| 7 | 7 | 25 | 6 | 1 | 9 | 5.34 |
| 8 | 8 | 30 | 6 | 3 | 11 | 11.66 |
| 9 | 9 | 35 | 8 | 2 | 9 | 5.51 |

**Supplementary Table S3**. Two-way ANOVA analysis of the main effects with coefficients that help in determining the significance of each parameter and also the model efficiency.

| **Source** | **Sum of squares** | **Degrees of freedom** | **Mean square** | ***F* value** | ***p*-value**  **Probability > *F*** |
| --- | --- | --- | --- | --- | --- |
| Model | 114.75 | 4 | 28.69 | 28.80 | 0.0033 |
| A-Temperature | 87.05 | 2 | 43.52 | 43.69 | 0.0019 |
| B-pH | 27.70 | 2 | 13.85 | 13.90 | 0.0158 |
| C-L-Asparagine | 0 | 0 | - | - | - |
| D-Glucose | 0 | 0 | - | - | - |
| Residual | 3.98 | 4 | 1.00 | - | - |
| Corrected total | 118.73 | 8 | - | - | - |

Predicted Activity (U mL^-1^)

Actual Activity (U mL^-1^)

**b)**

**a)**

L-Asparaginase activity (U mL^-1^)

Temperature


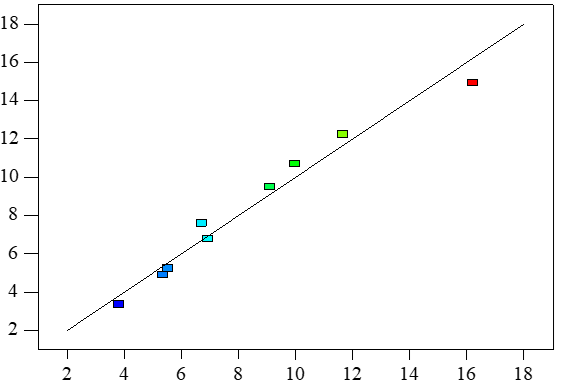


**Supplementary Figure S1.** a) Interaction plot between the two primary factors of temperature and pH showing the variation in the production of the enzyme. b) Taguchi model predicted versus experimentally obtained actual L-Asparaginase enzyme activity of Coprinopsis cinerea IBBLA4

**a)**

**b)**

**Supplementary Figure S2.** L-Asparaginase activity **(a)** and specific activity **(b)** of Trichosporon asahii IBBLA1 for varying inoculum concentration i.e. the number of cells used.

**Supplementary Figure S3:** The variation in the readings of UV-spectrophotometer for Blank (enzyme added after adding TCA), Test (enzyme kept for 30 min reaction) and Crude enzyme samples

**Taguchi Method of Optimization**

It is a technique that is used in the optimization of a process when different parameters have to be dealt and so most commonly used in the SmF process as it is simpler. It works on the combination of different parameters from as low as 2 parameters to as high as 63 different parameters to discuss the various effects of the individual parameters and their interactions. The optimum design is obtained for the improvement of the process. In this methodology, a study was done on the effect of 4 different parameters at 3 different levels to bring out the optimum result. The experiments for the required process is run and the result obtained is analysed using the data of ANOVA, where different values including F and p values give the confirmation of the significance of the selected model along the with the regression coefficient (R^2^).

One-factor-at-a-time technique was used to studied for the entire process and the most predominant in determining the activity of the enzymes was selected. 4 parameters (Temperature, pH, Carbon and Nitrogen concentration) was further analysed as a set using the Taguchi method of optimization to study the interaction effect of these parameters and the values are reported for the study to determine the significance of the model in understanding the whole process. Based on the obtained results, the model was found to be significant in analysing the data.

**Supplementary Figure S4.** L-glutaminase and urease activity for isolates showing positive presence of the L-glutaminase and urease enzyme
